# Crimean–Congo Hemorrhagic Fever Virus and *Borrelia burgdorferi* sensu lato in Ticks from Kosovo and Albania

**DOI:** 10.3389/fvets.2018.00038

**Published:** 2018-03-06

**Authors:** Kurtesh Sherifi, Agim Rexhepi, Kristaq Berxholi, Blerta Mehmedi, Rreze M. Gecaj, Zamira Hoxha, Anja Joachim, Georg G. Duscher

**Affiliations:** ^1^Faculty of Agriculture and Veterinary, University of Prishtina “Hasan Prishtina”, Pristina, Kosovo; ^2^Faculty of Veterinary Medicine, Agricultural University of Tirana, Tirana, Albania; ^3^Department of Pathobiology, Institute of Parasitology, University of Veterinary Medicine Vienna, Vienna, Austria

**Keywords:** Crimean–Congo hemorrhagic fever, Lyme borreliosis, *Hyalomma marginatum*, *Dermacentor marginatus*, *Ixodes ricinus*

## Abstract

Tick-borne diseases pose a serious threat to human health in South-Eastern Europe, including Kosovo. While Crimean–Congo hemorrhagic fever (CCHF) is a well-known emerging infection in this area, there are no accurate data on Lyme borreliosis and tick-borne encephalitis (TBE). Therefore, we sampled and tested 795 ticks. *Ixodes ricinus* (*n* = 218), *Dermacentor marginatus* (*n* = 98), and *Haemaphysalis* spp. (*n* = 24) were collected from the environment by flagging (all from Kosovo), while *Hyalomma marginatum* (*n* = 199 from Kosovo, all from Kosovo) and *Rhipicephalus bursa* (*n* = 130, 126 from Albania) could be collected only by removal from animal pasture and domestic ruminants. Ticks were collected in the years 2014/2015 and tested for viral RNA of CCHF and TBE viruses, as well as for DNA of *Borrelia burgdorferi* sensu lato by real-time PCR. In Kosovo, nine ticks were positive for RNA of Crimean–Congo hemorrhagic fever virus and seven for DNA of *B. burgdorferi* s. l. None of the ticks tested positive for TBEV. CCHF virus was detected in one *H. marginatum* male specimen collected while feeding on grazing cattle from the Prizren region and in eight *R. bursa* specimens (five females and three males collected while feeding on grazing sheep and cattle) from the Prishtina region (Kosovo). *B. burgdorferi* s. l. was detected in seven questing ticks (four male and one female *D. marginatus*, two *I. ricinus* one female and one male) from the Mitrovica region (Kosovo). Our study confirmed that CCHF virus is circulating in Kosovo mainly in *H. marginatum* and *R. bursa* in the central areas of the country. *B. burgdorferi* s. l. was found in its major European host tick, *I. ricinus*, but also in *D. marginatus*, in the north of the Kosovo. In order to prevent the spread of these diseases and better control of the tick-borne infections, an improved vector surveillance and testing of ticks for the presence of pathogens needs to be established.

## Introduction

Crimean–Congo hemorrhagic fever virus (CCHFV) is a tick-borne pathogenic member of the Bunyavirales order, Nairoviridae family, genus Orthonairovirus (1). CCHFV is present in different countries in Africa, Asia, and Europe (2). The occurrence of CCHFV in Europe corresponds with the circulation of *Hyalomma marginatum* ticks, which are both reservoirs and vectors of the virus (3).

In the southeast of Europe Crimean–Congo hemorrhagic fever (CCHF) is sporadic or endemic in Bulgaria, Kosovo, Albania, Greece, and Turkey (4–6). In Kosovo, 304 persons were diagnosed with CCHF with 21% case fatality rate between 1954 and 2014 (7). In about 50% of the Kosovo regions, CCHFV is widespread with hyperendemic areas in the municipalities of Malishevë, Rahovec, Suharekë, and Klinë (Prizren, Gjakove, and Peja regions). The prevailing CCHF season is between May and July, which corresponds with the peak activity of *H. marginatum* ticks. CCHF human cases in Albania occurred mostly in the region bordering Kosovo, respectively, in Kukes and Has municipalities (8).

In a study conducted by Sherifi et al. (9) who analyzed 1,102 ticks by RT-PCR, 3.6% were positive for CCHFV, mostly *H. marginatum* (9.8% or 29/297 tick samples in endemic areas) and in *Rhipicephalus bursa* (9.3% or 10/108 samples in non-endemic areas), while only in one, *Ixodes ricinus* viral RNA of CCHFV was detected. In 2013, Kosovo experienced an outbreak of CCHF in humans with 26 cases confirmed clinically and by RT-PCR, 13 of whom have died (10). In Albania, 385 *H. marginatum* in 73 pools and 277 *R. bursa* in 59 pools were sampled and tested in the years 2007–2010, and 11.8% of the pools of *R. bursa* were positive for CCHFV by RT-PCR (11).

In Kosovo and Albania, two strains of CCHF virus, Europe 1 and 2, are circulating (Clade V and VI). The strain Europe 1 (Clade V) appears to be transmitted by *H. marginatum* ticks and is highly pathogenic for humans, while the strain Europe 2 (Clade VI) is found exclusively in *R. bursa* ticks and is associated with the subclinical or mild cases of CCHF in humans (9, 11). Also, in Greece and Turkey, strain Europe 2 (Clade VI) was from ticks and humans (including the prototype strain AP92) (12, 13).

Lyme borreliosis is a widespread tick-borne disease caused by the pathogen *Borrelia burgdorferi* sensu lato and its predominant vector in Europe is *I. ricinus* (14). Borreliae are Gram-negative bacteria of the family *Spirochaetaceae*. *B. burgdorferi* s. l. can be divided into different genospecies, of which *B. afzelii, B. burgdorferi* sensu stricto, *B. garinii* and, possibly, *B. valaisiana* have pathogenic significance (15). *B. afzelii* is more widespread in the Northern, Central, and Eastern parts of Europe and *B. garinii* in the Western parts (16). The disease can manifest itself in a variety of disease forms (17, 18). About 65,000 human cases of Lyme borreliosis are reported annually in Europe (19). The prevalence of *Borrelia* in ticks in Europe is 13.7%, whereas the prevalence is higher in adults (18.6%) than in nymphs (10.1%) (20). In the city of Hannover (Germany), infection rates were 33.3% in adult *I. ricinus* ticks, 20.3% in nymphs, and 25.8% in larvae (21). In *I. ricinus* adults collected in Bulgaria, the prevalence of *B. burgdorferi* s. l. was almost 40%, while *B. afzelii* was the main species with >50% (22).

In Kosovo, no data about the prevalence of Lyme borreliosis in humans or *B. burgdorferi* in ticks are available. In the Clinic of Infectious Diseases at the University Clinical Center of Kosovo, 30 patients were diagnosed clinically by ELISA for Lyme borreliosis in 2015, thus confirming the presence of the pathogen in the area. A number of infections in Kosovo probably remain undiagnosed.

Tick-borne encephalitis (TBE) is a viral tick-borne disease that is present in endemic regions in different countries of Europe and Asia. TBEV (family *Flaviviridae*) encompass three subtypes, the Western subtype, which is predominant in central, eastern, and northern Europe; the Siberian subtype, endemic in eastern Europe, Russia, and northern Asia; and the Far Eastern subtype, endemic in eastern Russia and some parts of China and Japan (23, 24). *I. ricinus* is a vector of TBEV in central, northern, and eastern Europe and *I. persulcatus* (in northeastern Europe and northern and central Asia). Small rodents are the competent reservoir hosts of TBEV. Humans usually acquire the infection by tick bites and consumption of raw dairy products derived from infected animals (25). Several thousand cases with neuro-invasive illness are recorded in Europe and Asia annually, which also represent an international public health problem because of increasing mobility and travels to endemic regions (24). In Eastern Europe, TBE was reported from Slovenia, Croatia, Bosnia, Serbia, Albania, and Greece (24–26). TBEV prevalence in tick populations was determined for endemic regions like Austria (0.44–6.2%), Finland (0.07–2.57%), Germany (0.3–5.3%), and Slovenia (0.43%) (27–30). In Albania, 82 TBE cases (described as nonbacterial meningoencephalitis) were reported from 1983 to 1990 (31, 32). From 1990 to 1996, 67 cases were registered. Mostly people living in rural areas were infected by ingestion of unpasteurized milk and dairy products (Berxholi 2016, personal communication).

The aim of the study was to determine the tick species and their infection rates for CCHFV, *B. burgdorferi* s. l., and TBE, where especially for latter pathogen, no data on humans or ticks Kosovo are available so far, in different regions of Kosovo and Albania.

## Materials and Methods

### Study Area and Tick Collection

Kosovo has a total area of 10,900 km^2^, with approximately 40% fragmented agricultural land surrounded by 60% mountains with sea level between 276 and 2,656 m. The climate is Mediterranean continental and European continental (33). Albania borders Kosovo to the South-West has a total area of 28,748 km^2^ and 70% of the country is mountainous. The climate is typically Mediterranean except for the most north-western parts with high altitude areas (34). In Kosovo, ticks were collected in 15 villages of four regions (Prizren, Prishtina, Mitrovice, and Ferizaj) during April–June and September–October 2014–2015. In this area, a continental climate dominates, characterized by cold winters and hot summers. The maximum temperatures are reached during the summer, averaging +25°C, while the minimum temperatures are displayed in January, at an average value of −3°C. Atmospheric precipitation is at an average of 751 mm per year. The agricultural land is fragmented and the villages are surrounded by low mountains and have lands with bushes. The ticks were randomly collected in the localities with an altitude between 450 and 700 m. In Albania, ticks were collected in four villages of three different municipalities in the North-East (Kukes), the North-West (Lezhe), and the South of the country (Gjirokaster). Due to logistic constraints, the sample size did not reach the scheduled amount. However, *I. ricinus, Dermacentor marginatus*, and Haemaphysalis spp. were collected by flagging, while *H. marginatum* and *R. bursa* were sampled from the ground mostly on animal pastures or directly from livestock in order to remove ticks from the infested animals by the use of paired forceps. Latter two tick species are more likely to be found on host (35), consequently, sampling during feeding on host or on the pasture close to the hosts deliver more ticks in shorter time, which in turn makes comparison to flagged ticks almost impossible. To evaluate a possible spreading of CCHF virus from the known endemic areas Prizren and Prishtina to other neighboring regions (Mitrovica and Ferizaj) through the movement of domestic animals, we have selected randomly the sites for tick collection near domestic animals farm. During the tick collection, adequate personal protection was applied. Ticks were placed into 2 ml cryovials and stored at −80°C in the veterinary laboratory in Prishtina. Identification of tick species and sex determination were done under a stereomicroscope using the key of Estrada-Peña et al. (36).

### RNA/DNA Extraction from Ticks

The extraction was carried out under the same conditions and in the same facility as used for human samples tested for CCHFV. Single whole ticks were placed in a 2 ml Eppendorf tube containing three to five steel beads (7 mm, Qiagen, Hilden, Germany), frozen on liquid nitrogen, and directly granulated in a Tissue-Lyser LT (Qiagen, Hilden, Germany) with a −20°C pre-cooled rotor at 50 Hz for 2–5 min. Repeated freezing and lysis was necessary in some cases. The samples were then re-suspended in 300–500 µl PBS (depending on tick size) containing 10% fetal calf serum, 500 IU/ml penicillin, and 500 µg/ml amphotericin, and centrifuged at 2,000 × *g*. For viral RNA extraction, 200 µl of the supernatant was diluted in 200 µl distilled water including carrier RNA and proteinase K (RTP DNA/RNA Virus Mini Kit, Stratec, Birkenfeld, Germany), following sample inactivation for 10 min in 95°C and DNA/RNA extraction according to the manufacturer’s instructions.

### Reverse Transcriptase-PCR Detection for CCHFV RNA and TBEV RNA

For the first round of the RT-PCR, 2 µl RNA each of up to five single tick extractions were pooled (10 µl) to obtain 160 pools for detection of CCHF virus. Only for CCHF virus-positive pools, single tick diagnostic PCRs were subsequently conducted. The RT-PCRs for CCHFV (Europa 1 (Clades V) and Europa 2 (Clades VI) or variant AP92) were carried out using the RealStar^®^ CCHFV RT-PCR Kit 1.0 (Altona Diagnostics, Hamburg, Germany), according to the manufacturer’s instructions. For the detection of TBEV (European, Far Eastern, and Siberian viral subtypes) in ticks, 1 µl DNA/RNA eluates each of up to four single tick extractions were pooled (4 µl) to obtain a total of 200 pools and RT-PCR was performed using the ixSave^®^ TBE real-time RT-PCR Kit TM (Gerbion, Kornwestheim, Germany). Sequence amplification were performed and analyzed on a Rotor-Gene Q^®^ (Qiagen, Hilden, Germany).

### Quantitative Real-time PCR for *B. burgdorferi* s. l. DNA

For the detection of *B. burgdorferi* s. l., 1 µl DNA/RNA eluates each of up to four single tick extractions were pooled (4 µl) to obtain 200 pools and analyzed with ixSave^®^ Borrelia real time PCR Kit (Gerbion, Kornwestheim, Germany) on a Rotor-Gene Q^®^ (Qiagen, Hilden, Germany). Similar to the CCHFV procedure, only the single tick extracts of positive pools were further analyzed individually.

## Results

For both sampling strategies, only adult ticks could be collected. The most abundant questing tick species in Kosovo collected by flagging (*n* = 340) was *I. ricinus* (64.2%), followed by *D. marginatus* (28.8%) and *Haemaphysalis* spp. (7%) (Table 1). Of these, seven of them were positive for *B. burgdorferi* s. l., but none of them was positive for CCHFV and TBEV (Table 1).

**Table 1:** Ticks and infections rates for *Borrelia burgdorferi* s. l. and Crimean–Congo hemorrhagic fever virus (CCHFV) in different regions of Kosovo.

Ticks	Sample collection	*N* ticks	Mitrovice	Prishtina	Prizren	Ferizaj	Total	Detection of positive individuals
*Ixodes ricinus*	Vegetation	Total	134	29	16	29	195	
	Vegetation	Females	67*	23	15	19	124	*B. burgdorferi (*N* = 1)
	Vegetation	Males	54*	6	1	10	71	*B. burgdorferi (*N* = 1)
		Subtotal	134	29	16	39	218	

*Dermacentor marginatus*	Vegetation	Total	98	0	0	0	98	
	Vegetation	Females	44*	0	0	0	44	*B. burgdorferi (*N* = 1)
	Vegetation	Males	54*	0	0	0	54	*B. burgdorferi (*N* = 4)
		Subtotal	98	0	0	0	98	

*Haemaphysalis* spp.		Total	20	4	0	0	24	
	Vegetation	Females	8	2	0	0	10	
	Vegetation	Males	12	2	0	0	14	
		Subtotal	20	4	0	0	24	

*Hyalomma marginatum*	Field	Total	0	0	132	0	132	
	Field	Females	0	0	66	0	66	
	Field	Males	0	0	66	0	66	
	Host	Total	7	47	9	4	67	
	Host	Females	0	15	1	0	16	
	Host	Males	7	32	8*	4	51	*CCHFV (*N* = 1)
		Subtotal	7	47	141	4	199	

*Rhipicephalus bursa*	Field	Total	0	0	0	0	0	
	Field	Females	0	0	0	0	0	
	Field	Males	0	0	0	0	0	
	Host	Total	0	116	0	14	130	
	Host	Females	0	60*	0	12	72	*CCHFV (*N* = 5)
	Host	Males	0	56*	0	2	58	*CCHFV (*N* = 3)
		Subtotal	0	116	0	14	130	

*Detection of positive individual ticks for B. burgdorferi s. l. and CCHFV.

*Hyalomma marginatum* (*n* = 199 from Kosovo) and *R. bursa* (*n* = 130 from Kosovo and 126 from Albania) that are competent vector for CCHFV could not be sampled by flagging; instead, 132 *H. marginatum* were picked from the ground or from animal pasture and 67 were collected from domestic ruminants. One of the latter (*H. marginatum)* was positive on CCHFV. Of the 130 tested *R. bursa* ticks from Kosovo collected from domestic animals, eight were positive for CCHFV (Table 1).

In Albania, none of the 126 collected *R. bursa* ticks was positive for the investigated pathogens.

Overall, CCHFV RNA was detected in one *H. marginatum* and eight *R. bursa* specimens (total of 9 ticks) collected while feeding on nine different individual animals (6 cattle and 3 sheep). In the Prizren region (village Senike, Malishevë municipality), one male of nine *H. marginatum* ticks collected from grazing cattle in April 2014 was positive. In the second region of Kosovo where CCHF virus-positive ticks were found, Prishtina, all eight positive *R. bursa* while feeding (five female and three male) were collected from grazing sheep and cattle in June 2014.

*Borrelia burgdorferi* s. l. was detected in seven of the 340 flagged ticks (2.0%) from Kosovo and was found only in the Mitrovica region (northern Kosovo). Two of 134 *I. ricinus* (1.5%) and five of 98 *D. marginatus* (5.1%), collected in May 2014 by flagging tested positive for *B. burgdorferi* s. l. None of the ticks (*H. marginatum, R. bursa*) collected from the pastures or animals were positive for this pathogen.

## Discussion

Crimean–Congo hemorrhagic fever virus is present on the Balkan Peninsula, and at least in some regions it appears to be closely linked to the presence of *H. marginatum* ticks as its main vector and reservoir (37). The detection of CCHFV in *H. marginatum* ticks from the southwestern Prizren region and in *R. bursa* from the northeastern Prishtina region of Kosovo, which are the dominant tick species in the respective regions, provides strong evidence of the continuous presence of CCHFV in the country. In our study, nine of tested ticks were positive for viral RNA of CCHFV, although based on the low amount of ticks sampled, any conclusions on the actual risk has to be done with caution. Additionally, it has to be stated that due to the different sampling method, no real prevalence data can be drawn for CCHFV. But, the results indicate the circulation of this virus in particular on these locations. For a more accurate estimation, a higher number of ticks has to be investigated. The Prizren region encompasses the municipalities of Malishevë, Rahovec, Suharekë, and Klinë, which are known endemic regions for CCHF. In the last CCHF disease outbreak in this region in 2013, 13 out of 26 patients clinically and laboratory diagnosed with CCHF died (10). In the present study, one *H. marginatum* tick out of nine collected in the village of Senik, Malishevë municipality, harbored CCHFV RNA. Sherifi et al. (9) collected *H. marginatum* ticks in Malishevë municipality in 2012 and found 8.6% to be positive for CCHFV. In the Klinë municipality, 4% of the ticks harbored RNA of CCHFV, seven *H. marginatum*, and one *I. ricinus*. Malishevë, Klinë, Suharekë, and Rahovec municipalities are the CCHF endemic areas in Kosovo, and every 3–5 years, disease outbreaks with high mortality rates are recorded. The most affected groups are agricultural workers and animal farmers.

The highest amount of CCHFV-positive ticks in the Prishtina region occurred in the village of Hajvali, with 8 of 115 tested *R. bursa*. In this region, no human cases of CCHF have been reported so far, which may be due to a lower affinity of *R. bursa* to humans compared to *H. marginatum*, or the presence of different CCHFV strains. Nevertheless, both tick species represent competent vectors and are of enormous importance in order to estimate any risk outgoing from those ticks. Interestingly, flagging for these two species is known to be less effective (35, 38), and sampling from the hosts is the method of choice to obtain those ticks (38–41). Collection of ticks from the hosts is sometimes used for other tick species as well (42, 43) although environmental collection closer reflects the real situation and, therefore, should be preferred (44). Anyhow, the collection and analysis of *R. bursa* and *H. marginatum* is of enormous interests and can be easily done from the hosts, but the abundance cannot directly be compared with the flagged tick samples. Hence, cross-over contamination of the animal blood cannot be excluded. However, analyzing these two species from Kosovo may reveal two CCHFV strains. One is “Kosova Hoti,” which was also isolated from the humans during the outbreak in Kosovo in 2001. It belongs to the Europe/Turkey group with high similarities to the Russian isolate “Drosdov strain.” This strain clusters with Europa 1 (Clade V) and is highly pathogenic for humans, and the competent biological vector is assumed to be *H. marginatum*. The second CCHFV strain is circulating between *R. bursa* and animals in Kosovo and is related to a strain isolated in the Greece (AP92). This strain clusters with Europe 2 (Clade VI) and is low pathogenic or non-pathogenic for humans (9, 45). A sero-prevalence study in south Albania and the Former Yugoslav Republic of Macedonia (FYROM) for the detection of anti-CCHF IgG antibodies in domestic ruminants revealed a high rate of sero-positivity of 23% in Albania and 49% in FYROM (46), indicating that CCHFV is circulating in domestic animals and ticks in these two countries.

The presence of CCHFV in ticks in Kosovo and Albania and the local outbreaks in humans every 3–5 years in southwestern Kosovo and North Albania are a strong indication that a CCHFV “high pathogenic” strain persists in those regions and correlates with the abundance of *H. marginatum* ticks. The circulation of a CCHFV “low” or “nonpathogenic” strain in northeastern Kosovo (near Prishtina) and in southern Albania correlates with the distribution of *R. bursa* ticks. We could not detect CCHFV neither in ticks from Mitrovica and Ferizaj, which are neighboring regions of Prizren and Prishtina, nor in ticks from Albania. The possibility of the spread of CCHFV into other regions and countries is very high, taking into account the movements of domestic and wild animals, as well as migratory birds. In the present study, testing of ticks near the endemic CCHF regions in Kosovo, namely in Mitrovice and Ferizaj, indicates that the distribution of *H. marginatum* in these two regions is very scarce, and none of them was infected with CCHFV. The spreading of infected *H. marginatum* females attached to domestic animals to another region can occur at any time in Kosovo and neighboring countries, but it of course then depends on the tick’s ability to adapt to new environmental conditions and to continue its life cycle.

The spread of CCHFV in different regions is possible through the migratory birds, which can carry infected ticks. The detection of CCHFV in such animals in Morocco indicates a risk of spreading the virus to Europe, and this assumption is also supported by the detection of the first human cases of CCHF in Spain in August 2016 (47, 48).

For further studies and in order to prevent the spread of this dangerous infectious agent, it will be very important to survey a possible re-assortment (49) of the two different CCHFV strains circulating in Kosovo and the neighboring countries and its role in the pathogenicity for humans in combination with tick surveillance and control, treatment of domestic animals with acaricides in endemic regions, as well as the detection of CCHFV RNA in ticks.

Lyme borreliosis is present in Kosovo and is related to the distribution of *I. ricinus* and *D. marginatus* ticks. Epidemiological data for infections of humans do not exist in the country due to the absence of a monitoring program. In the present study, 2% of 340 flagged tested ticks were positive for *B. burgdorferi* s. l. and found only in the Mitrovice region, were the most abundant ticks species were *I. ricinus* and *D. marginatus*. Of the 134 collected, *I. ricinus* 1.5% and the 98 *D. marginatus* 5.1% were positive for *B. burgdorferi* s. l. *D. marginatus* might play a role as a secondary vector in some ecosystems regarding *B. burgdorferi* s. l., as stated from Bulgaria (50), but this has to be proven with transmission studies.

In the present study, TBEV was not detected in ticks from Kosovo or Albania, probably because even in highly endemic areas, TBEV distribution follows a scattered and very focal pattern (51). Transmission foci are mostly restricted to small areas and the detection of these foci is limited by the numbers of ticks sampled as well as the sampling locations. For accurate determination of TBEV foci, a very small meshed sampling design is needed (52). Additionally, the virus load in ticks can be below the detection limit (53); therefore, the lack of TBEV in the ticks in this study does not confirm the absence of TBEV in the sampling area.

This study showed that the distribution of tick-borne pathogens is frequently linked to the distribution of the main tick vector species, and surveillance of these pathogens must include the determination and examination of putative vector species in endemic and neighboring regions in order to prevent the spread of diseases. In Kosovo (and possibly also in Albania where only comparatively few ticks could be sampled), there is an eminent risk to contract CCHV and *B. burgdorferi* s. l. by tick bites, but the distribution of the pathogens and their vectors is uneven and detailed risk analyses require more detailed information on their distribution.

## Author Contributions

KS, AJ, and GD designed the study. AR, BM, KB, and ZH conducted the fieldwork. KS, RG, and GD conducted the laboratory work. All the authors contributed to the manuscript editing and approved the final manuscript.

## Conflict of Interest Statement

The authors declare that the research was conducted in the absence of any commercial or financial relationships that could be construed as a potential conflict of interest.
